# Supplementary material for: Nitrogen limitation reveals large reserves in metabolic and translational capacities of yeast
Source: Nat Commun. 2020 Apr 20;11:1881. doi: 10.1038/s41467-020-15749-0 (PMC7171132; doi:10.1038/s41467-020-15749-0)
Supplement: Supplementary file 1 — Supplementary Information [file 41467_2020_15749_MOESM1_ESM.pdf]

Supplementary Information

Nitrogen limitation reveals large reserves in metabolic and translational capacities of yeast

Yu et al.

Supplementary Methods

Sample Preparation for Proteomic Analysis

Yeast cell pellets were resuspended in lysis buffer containing 2% sodium dodecyl sulfate and 50 mM triethylammonium bicarbonate in water, and homogenized using a FastPrep®-24 instrument (Matrix D (green-capped tubes); MP Biomedicals, OH, USA) for 5 repeated 40 seconds cycles at 6.5 m/s, with 30-60 seconds pause between. The lysate was transferred to new tubes, diluted 10 times with the lysis buffer and protein concentration was determined using Pierce™ BCA Protein Assay (Thermo Scientific) and the Benchmark Plus microplate reader (BIO-RAD) with BSA solutions as standards. The pooled Reference sample was prepared from the aliquots of the lysates of S. cerevisiae CEN.PK 113-7D cells from the J. Nielsen Lab (Chalmers, Gothenburg, Sweden).

For the TMT-based relative quantification, aliquots containing 30 µg of protein were taken from each experimental sample and from the pooled Reference sample; an aliquot of 50 µg of the pooled sample was spiked with 10.6 µg of the UPS2 Proteomics Dynamic Range Standard (Sigma-Aldrich, Saint-Louis, MO) for the IBAQ [1] quantification. Each sample was reduced by addition of 2M DL-Dithiothreitol (DTT) to a final concentration of 100 mM and incubated at 56°C for 30 min. The samples were processed according to the filter-aided sample preparation (FASP) method [2] with small modifications. In short, reduced samples were diluted to 300 µl by addition of 8M urea, transferred onto Nanosep 30k Omega filters (Pall Life Sciences) and washed 2 times with 200 µl of 8M urea. Alkylation of the reduced cysteine side chains was performed with 10 mM methyl methanethiosulfonate (MMTS) diluted in digestion buffer (1% sodium deoxycholate (SDC), 50 mM TEAB) for 30 min at room temperature and the filters were then repeatedly washed with digestion buffer. Trypsin in digestion buffer was added (300/500 ng, mass ratio 1:100) and the sample was incubated at 37°C for 4h, then another 1:100 portion of trypsin was added and incubated overnight. Digested peptides were collected by centrifugation at 11 600 xg for 20 min, followed by a wash with 20 µl of the digestion buffer and centrifugation at 11 600 xg for 20 min. Peptide samples for relative quantification were labelled using the TMT 10plex™ isobaric reagents according to the manufacturer's instructions (Thermo Scientific), combined into one TMT set, concentrated using vacuum centrifugation and SDC was removed by acidification with 10% TFA and subsequent centrifugation at 16 200 xg for 10 min.

The TMT-sets were fractionated into 44 primary fractions by basic reversed-phase chromatography (bRP-LC) using a Dionex Ultimate 3000 UPLC system (Thermo Fischer Scientific, Waltham, MA, USA).

Peptide separations were performed using a reversed-phase XBridge BEH C18 column (3.5 µm, 3.0x150 mm, Waters Corporation) and a linear gradient from 3% to 40% solvent B over 17 min followed by an increase to 100% B over 5 min. Solvent A was 10 mM ammonium formate buffer at pH 10.0 and solvent B was 90% acetonitrile, 10% 10 mM ammonium formate at pH 10.00. The primary fractions were concatenated into final 20 fractions (1+21+41, 2+22+42, … 20+40), evaporated and reconstituted in 15 µl of 3% acetonitrile, 0.2% formic acid for nLC MS analysis.

The pooled sample for the label-free quantification was fractionated into 40 primary fractions that were concatenated into 10 final fractions (1+11+21+31, 2+12+22+32, etc). The final fractions were evaporated and reconstituted in 15 µl of 3% acetonitrile, 0.2% formic acid for nLC MS analysis.

SpikeTides TQ synthetic peptide standards were purchase from JPT Peptide Technologies (Berlin, Germany). Two dried aliquots for each peptide were dissolved in two solvents: in 10% acetonitrile, 25 mM triethylammonium bicarbonate (TEAB), and in 0.5% aqueous sodium deoxycholate (SDC), 25 mM TEAB solution. Aliquots containing 100 pmol of each peptide were and each dissolution were combined into two standard peptide mixtures, according to the solvent used for solubilization. The peptide mixtures were digested with 2.5 µg of trypsin (Pierce Trypsin Protease, MS Grade, Thermo Fisher Scientific) for 2 h at 37°C, then another 2.5 µg of trypsin was added and the digestion was continued for 2 h at 37°C. The digested peptide samples were diluted to 800 µl with the solvent, 50 mM TEAB/10% acetonitrile for dissolution 1 and 50 mM TEAB, 0.5% SDC, and the equal aliquots form each mixture were labelled with two of the TMT 10plex™ isobaric reagents according to the manufacturer's instructions (Thermo Scientific). The labelled samples were combined into one TMT set, concentrated using vacuum centrifugation and SDC was removed by acidification with 10% TFA and subsequent centrifugation at 16 200 xg for 10 min. The peptides in the supernatant were purified using the Pierce C18 Spin Columns (Thermo Scientific) according to the manufacturer's instructions. The purified peptide solution eluted from the C18 spin column was dried on Speedvac and reconstituted in 15µl of 3% acetonitrile, 0.2% formic acid for the data-dependent LC-MS/MS analysis. The dissolution in 0.5% SDC/25 mM TEAB was found to yield higher relative abundance for some of the standards, and thus it was chosen for the subsequent experiments.

For the absolute quantification experiment a 30 µg aliquot of the pooled reference sample was digested using the FASP protocol as described above, with the following changes: 0.5% SDC/50 mM TEAB digestion buffer was used, the sample was digested for 2 h with 1.5 µg of trypsin (1:20), followed by addition of 1.5 µg of trypsin and digestion for 4 h. The digested sample was split into the aliquots containing 5.0 µg and 25 µg of total peptides, the volume of the smaller aliquot was topped up with the digestion buffer to match the volume of the bigger aliquot, and the aliquots were

labelled with the TMT 10plex™ reagents 126 and 127N according to the manufacturer's instructions. The digested mixture of the standard peptides from the 0.5% SDC/25 mM TEAB dissolutions was aliquoted into four samples that contained 125 fmol, 500 fmol, 2.5 pmol and 12.5 pmol of each standard peptide, respectively. The volumes of the smaller aliquots were equalized to 100 µl using the digestion buffer, and the aliquots were labelled the TMT 10plex™ reagents 128C, 129N, 130C and 131. The labelled cellular and standard samples were combined into one TMT set, concentrated using vacuum centrifugation and SDC was removed by acidification with 10% TFA and subsequent centrifugation at 16 200 xg for 10 min. The peptides in the supernatant were purified using the Pierce C18 Spin Columns (Thermo Scientific). The purified peptide solution eluted from the C18 spin column was dried on Speedvac and reconstituted in 15µl of 3% acetonitrile, 0.2% formic acid for the LC-MS/MS analysis.

Liquid Chromatography-Mass Spectrometry Analysis

All samples were analyzed on an Orbitrap Fusion Tribrid mass spectrometer interfaced with Easy-nLC1200 liquid chromatography system (both Thermo Fisher Scientific, Waltham, MA, USA). Peptides were trapped on an Acclaim Pepmap 100 C18 trap column (100 µm x 2 cm, particle size 5 µm, Thermo Fischer Scientific) and separated on an in-house packed analytical column (75 µm x 30 cm, particle size 3 µm, Reprosil-Pur C18, Dr. Maisch) using the linear gradients with 0.2% formic acid in water as a solvent A and 80% acetonitrile, 0.2% formic acid as solvent B with the flow of 300 nL/min.

Each TMT-labelled fraction for relative quantification analysis was separated on a gradient 6% to 35% B over 45 min, from 35% to 100% B over 5 min and 100% B for 10 min. Each fraction for the label-free IBAQ quantification was analyzed 3 times using the gradient 5% to 35% B over 75 min, from 35% to 100% B over 5 min and 100% B for 10 min. The TMT-labeled samples for the absolute quantification using the synthetic standards were separated on a gradient 5% to 40% B over 120 min, from 40% to 100% B over 10 min and 100% B for 10 min.

For the TMT-based relative quantification, MS scans were performed at 120 000 resolution, m/z range 380-1400 with the wide quadrupole isolation and AGC target 4e5; the most abundant precursors with charges 2 to 7 were selected for fragmentation over the 3s cycle time with the dynamic exclusion duration of 45 s. Precursors were isolated with a 0.7 Da window, fragmented by collision induced dissociation (CID) at 35 collision energy with a maximum injection time of 50 ms and AGC target 1e4, and the MS^2 spectra were detected in the ion trap followed by the synchronous isolation of the 7 most abundant MS^2 fragment ions within m/z range of 400-1400, and

fragmentation by higher-energy collision dissociation (HCD) at 60%; the resulting MS^3 spectra were detected in the Orbitrap at 50,000 resolution with m/z range 100-500, maximum injection time 100 ms and AGC target 1e5.

For label-free quantification IBAQ experiment, MS scans were performed at 120 000 resolution, m/z range 380-1480 with the wide quadrupole isolation and AGC target 4e5; the most abundant precursors with charges 2 to 7 were selected for fragmentation over the 1s cycle time with the dynamic exclusion duration of 40 s. Precursors were isolated with a 1.0 Da window, fragmented by collision induced dissociation (CID) at 35 collision energy with a maximum injection time of 40 ms and AGC target 1e4, and the MS^2 spectra were detected in the ion trap.

For the absolute quantification experiments, the sample that consisted only of the TMT-labelled synthetic peptides was analysed with the MS scans at 120 000 resolution, m/z range 350-1600 with the wide quadrupole isolation and AGC target 4e5; the most abundant precursors with charges 2 to 7 were selected for fragmentation over the 3s cycle time with the dynamic exclusion duration of 9 s. Precursors were isolated with a 0.7 Da window, fragmented by collision induced dissociation (CID) at 35 collision energy with a maximum injection time of 50 ms and AGC target 2e4, and the MS^2 spectra were detected in the ion trap followed by the synchronous isolation of the 5 most abundant MS^2 fragment ions within m/z range of 400-1400, and fragmentation by higher-energy collision dissociation (HCD) at 65%; the resulting MS^3 spectra were detected in the Orbitrap at 30,000 resolution with m/z range 100-500, maximum injection time 60 ms and AGC target 5e4. The standard data was processed as described below, and the m/z and the retention time values were exported as a mass list for the absolute quantification analysis. The TMT-set that contained the cellular yeast samples and the synthetic peptide standards was analysed with the MS scans at 120 000 resolution, m/z range 350-1570 with the wide quadrupole isolation and AGC target 4e5; the most abundant precursors from the targeted mass inclusion list were selected over the 5 s cycle time without dynamic exclusion. Precursors were isolated with a 0.7 Da window, fragmented by collision induced dissociation (CID) at 35 collision energy with a maximum injection time of 100 ms and AGC target 2e4, and the MS^2 spectra were detected in the ion trap followed by the synchronous isolation of the 5 most abundant MS^2 fragment ions within m/z range of 400-1400 (or 480-1400 for the repeated analysis), and fragmentation by higher-energy collision dissociation (HCD) at 65%; the resulting MS^3 spectra were detected in the Orbitrap at 60,000 resolution with m/z range 100-500, maximum injection time 250 ms and AGC target 1e6.

The mass spectrometry proteomics data has been deposited to the ProteomeXchange Consortium via the PRIDE [3] partner repository with the dataset identifiers PXD12803 for the IBAQ data set,

PXD014962 for the TMT-based relative quantification data set and PXD015025 for the absolute quantification experiment with the standard peptides.

Proteomic Data Analysis

Peptide and protein identification and quantification was performed using Proteome Discoverer version 2.2 (Thermo Fisher Scientific) with Mascot 2.5.1 (Matrix Science, London, United Kingdom) as a database search engine. The baker's yeast (*Saccharomyces cerevisiae* ATCC 204508 / S288c) reference proteome database was downloaded from Uniprot (February 2018, 6049 sequences) and used for the database search on the TMT-based relative quantification files; the concatenated database containing the yeast sequences and the 48 UPS protein sequences was used for the processing of the UPS2-spiked files; the sequences of the synthetic peptides were supplemented with porcine trypsin sequence (52 sequences in total) for the database searches on the TMT-based absolute quantification using the synthetic peptides.

For the TMT relative quantification data and the label-free IBAQ data, trypsin with 1 missed cleavage was used as a cleavage rule, MS peptide tolerance was set to 5 ppm and MS^2 tolerance for identification was set to 600 mmu. Variable modifications of methionine oxidation, and fixed modifications of cysteine methylthiolation were used for both sub-data sets. TMT-6 label on lysine and peptide N-termini was set as a fixed modification for the TMT-data. Percolator was used for the peptide-spectrum match (PSM) validation with the strict false discovery rate (FDR) threshold of 1%.

Precursor ion quantification was accomplished via the Minora feature detection node in Proteome Discoverer 2.2, with the maximum peak intensity values used for quantification. For ribosomal proteins, when considering paralogs separately, both unique and shared peptides were used for IBAQ calculation. When paralogs were summed, shared peptides were added only once. For all other proteins, only unique peptides were considered for the IBAQ calculation. Abundances from the 3 technical replicates were averaged and divided by the number of theoretically observable peptides for a protein to yield the IBAQ intensity (the number of observable peptides being calculated using an in-house Python script). The known absolute amount values of the UPS2 standard proteins were used to scale the Log-transformed IBAQ intensity values versus Log-transformed protein concentration. Only UPS2 proteins with at least 2 identified unique peptides were used for scaling.

The TMT reporter ions were identified in the MS^3 HCD spectra with a mass tolerance of 3 milli mass units (mmu), the signal-to-noise (S/N) abundances of the reporter ions for the unique peptides were used for relative quantification. In case of the TMT-labeled data set for the relative quantification,

the resulting reporter abundance values for each sample were normalized within Proteome Discoverer 2.2 on the total peptide amount.

In case of the TMT data set for the absolute quantification, the reporter abundances of each peptide from the cellular samples and the spiked standard samples were processed manually. Due to the fact that the dynamic exclusion was not used in the experimental method, each identified peptide was associated with multiple quantification spectra. The quantification spectra for each standard peptide were then inspected manually, and the ′′best quality′′ quantitative result was selected for each peptide, based on two factors: 1) the average reporter S/N should be as high as possible and 2) there should be no abundant unassigned signals in the MS^2 spectrum, which corresponds to the given MS^3 quantitative spectrum. The multinotch synchronous precursor selection was set at the 5 most abundant MS^2 fragment ions, thus the 5 most abundant fragments were inspected in each MS2 spectrum of interest. The quantitative result for the ′′best quality′′ MS^3 spectrum was assigned as a quantitative result for the corresponding peptide. Then, the S/N abundances and the known amounts in the TMT channels that corresponded to the synthetic standards were used to calculate the Linear regression slope (intercept was set to 0) for each peptide (LINEST function in Microsoft Office Excel 2010); the linear regression slope and the S/N abundance from the cellular yeast channel from the SAME ′′best quality′′ quantitative spectrum was used to calculate the amount of the corresponding peptide in the cellular yeast sample.

Supplementary Figures

a

Condition	Glucose·H_2O (g/L)	$(NH_4)_2SO_4$ (g/L)
C-lim	7.5	5
C/N=30	7.5	0.5
C/N=50	7.5	0.29
C/N=115	7.5	0.125

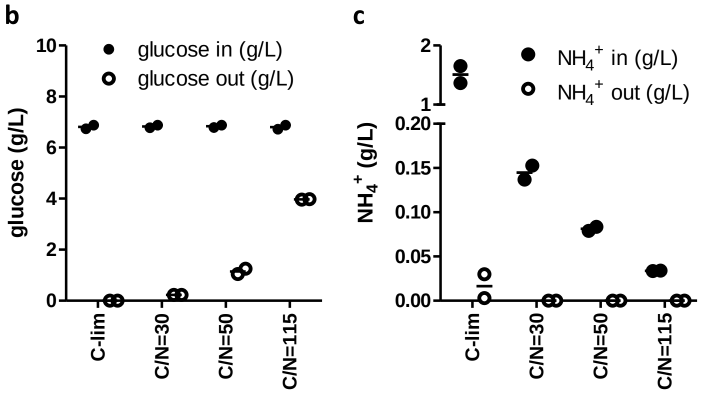

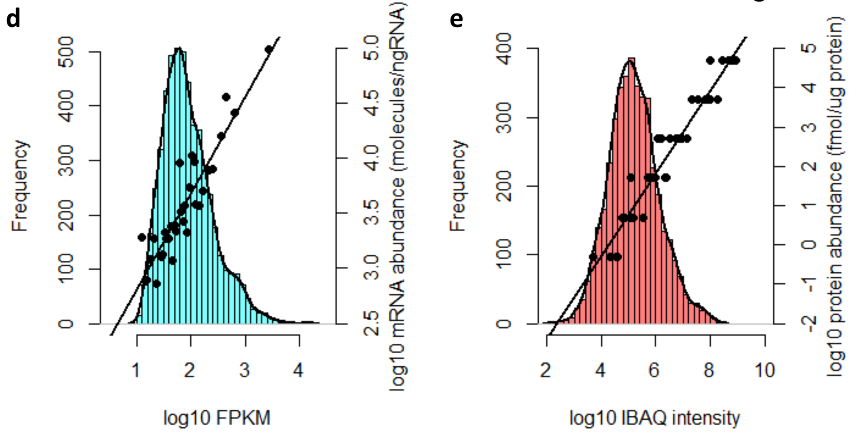

Supplementary Fig. 1. Parameters for the absolute quantification of transcript and protein abundance in yeast steady state cultures.

a. Media composition of chemostats.
b. Glucose content in chemostat medium and exhaust. Mean of biological duplicates are shown.
c. NH_4^+ content in chemostat medium and exhaust. Mean of biological duplicates are shown.
d. Distribution of the absolute mRNA abundance measured. The calibration curve using known concentrations of 31 transcripts is shown. The adjusted R^2 of the linear model was 0.84, p = 2.6e-13.
e. Distribution of the absolute protein abundance measured. The calibration curve using Proteomics Dynamic Range Standard (UPS2) is shown. The adjusted R^2 of the linear model was 0.95, p = 2.2e-16.

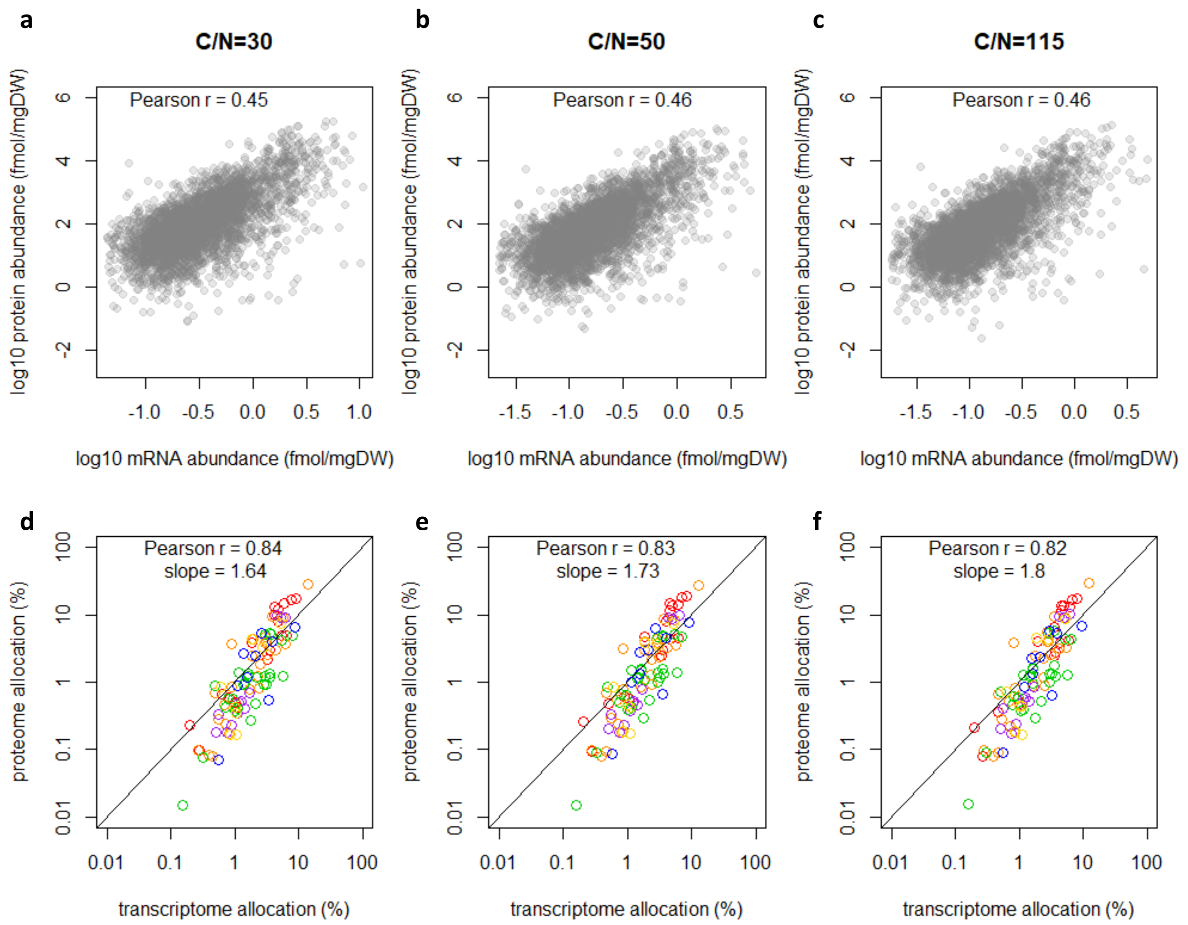

Supplementary Fig. 2. Transcript and protein abundance are poorly correlated in all conditions, but process-level allocation are well correlated.
a-c. Mean values of biological duplicates for absolute mRNA and protein abundance in N-limited chemostats.
d-f. The % (mol/mol) of total transcripts and proteins belonging to 99 GO-slim processes in N-limited chemostats were calculated. GO-slim terms are color-coded by functional category as in Fig 1b. Number of genes in each process can be found in Supplementary Data 2.

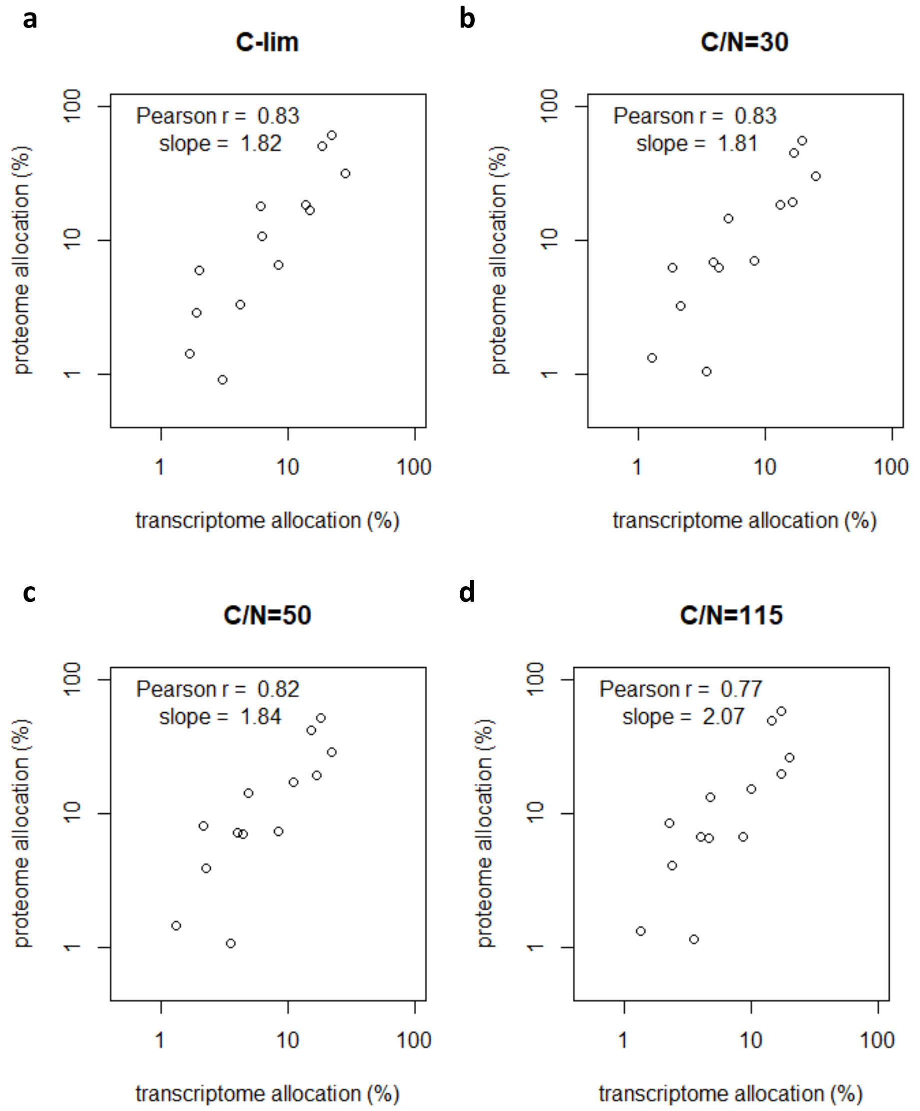

Supplementary Fig. 3. Transcriptome and proteome allocation are well correlated by gene category. a-d. Transcriptome and proteome allocations to 13 gene categories (as defined by Metzel-Raz *et al.*, 2017 [4]) were calculated as a % (mol/mol) of total transcripts and proteins. Mean values of biological duplicates are shown. The Pearson correlation coefficient and slope are shown.

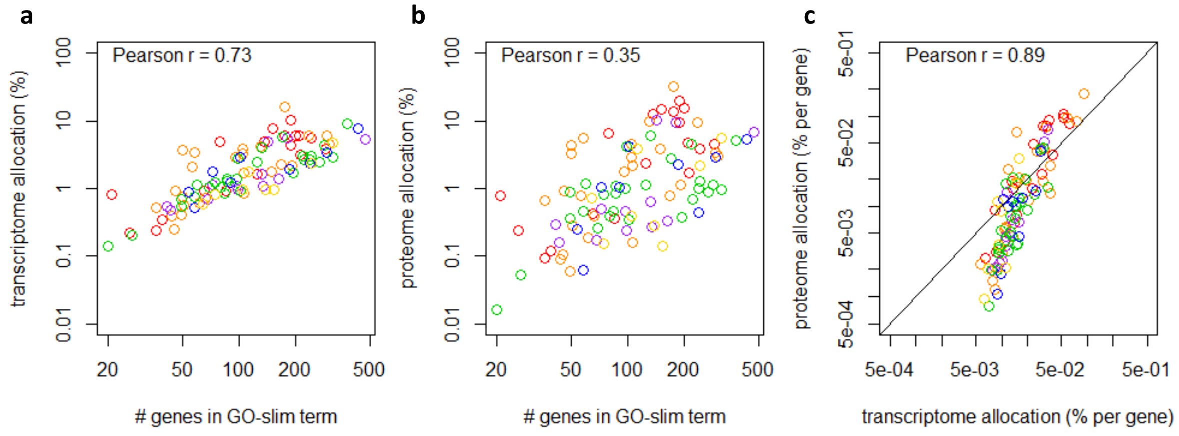

Supplementary Fig. 4. Transcriptome allocation is dependent on the number of genes in the process, but proteome allocation is independently regulated.

a. The % (mol/mol) of total transcripts belonging to 99 GO-slim terms in C-limited chemostats is plotted against the number of genes in the GO-slim term. GO-slim terms are color-coded by functional category as in Fig 1b.
b. As in (a) but for proteins
c. The % (mol/mol) of transcripts and proteins belonging to 99 GO-slim terms were normalized against the # of genes in each term. GO-slim terms are color-coded by functional category as in Fig 1b.

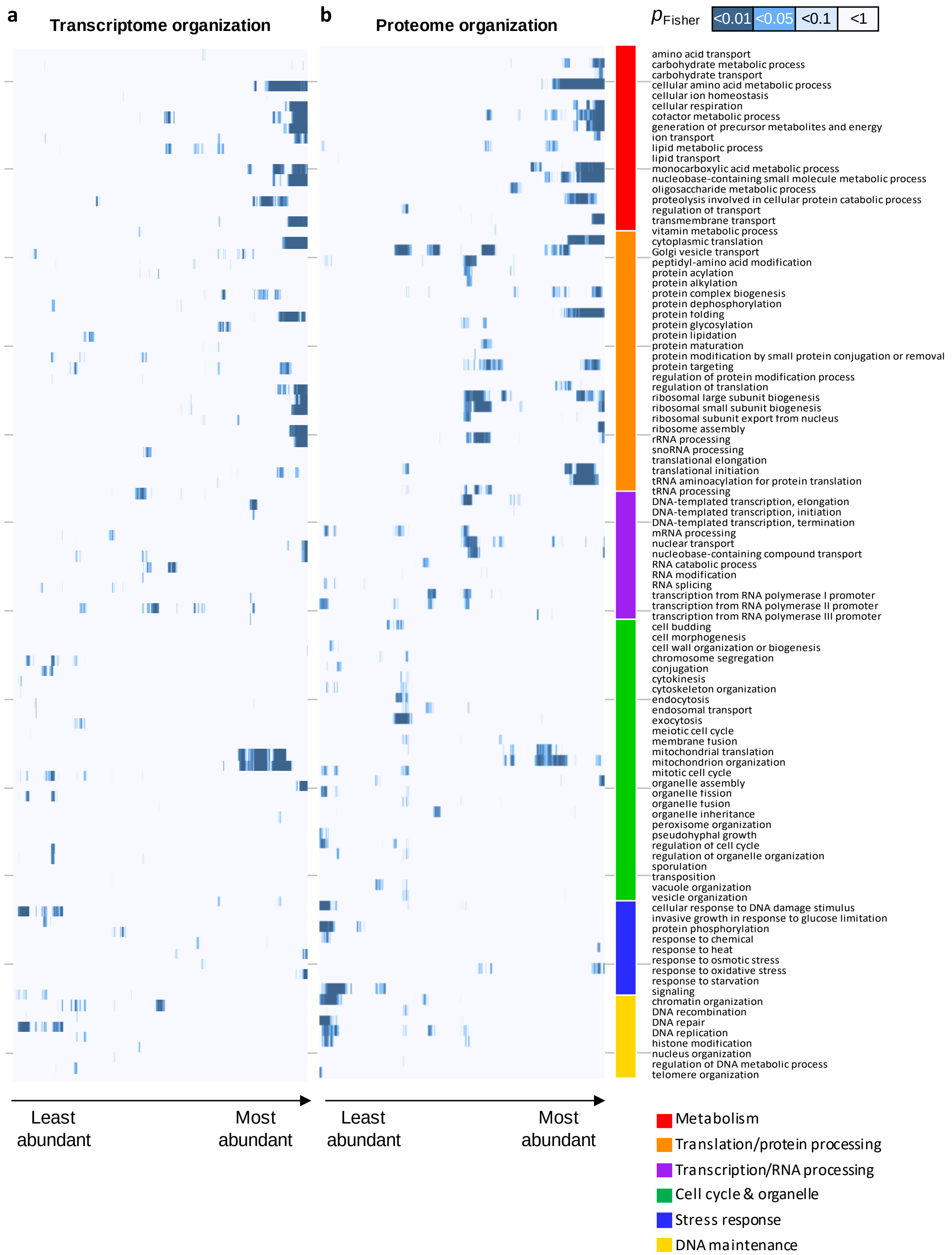

Supplementary Fig. 5. Transcriptome and proteome are organized by functional categories.

a. Enrichment of GO-slim terms in 200-gene sliding windows of relative transcript abundance was analyzed by two-sided Fisher's exact test. FDR-adjusted p_{Fisher} is color-coded as indicated. GO-slim terms are color-coded by functional category as indicated. Number of genes in each process can be found in Supplementary Data 2.

b. As in (a) but for protein abundance.

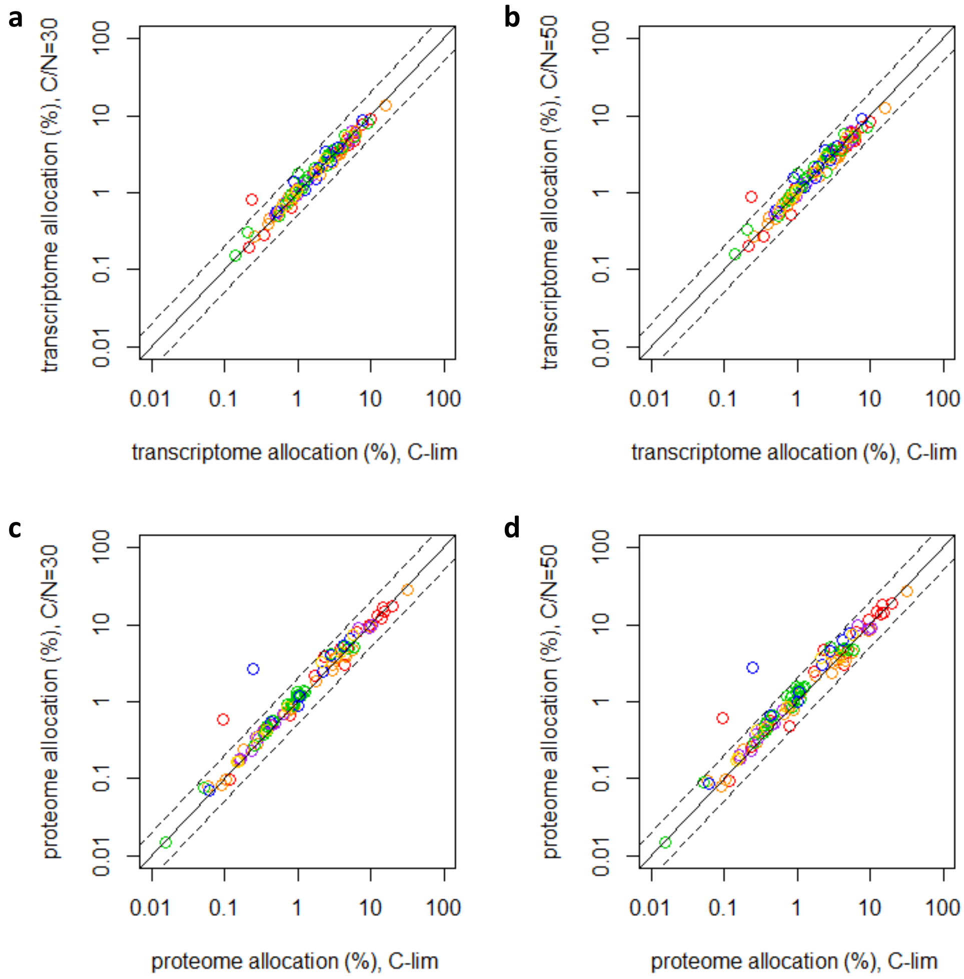

Supplementary Fig. 6. Transcriptomic and proteomic allocation are constant with respect to the growth rate.

a-b. Transcriptome allocation to 99 GO-slim terms were compared between N-limited and C-limited cultures. GO-slim terms are color-coded by functional category as in Fig 1b. Dashed lines are 2-fold increase/decrease from y=x, solid line. Mean values of biological duplicates are shown. The red outliers represent the “amino acid transport” GO-slim term.

c-d. As in (a-b) for proteome allocation. The red and blue outliers represent “amino acid transport” and “response to starvation” GO-slim terms, respectively.

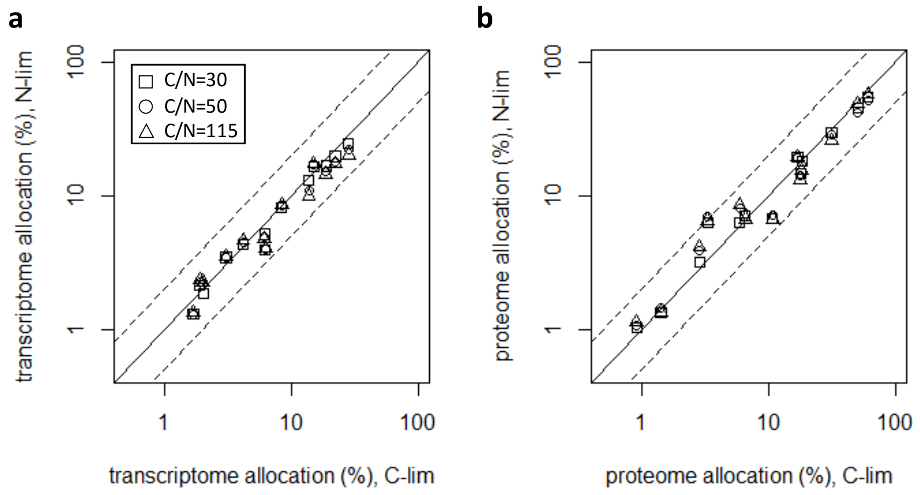

Supplementary Fig. 7. Process-level transcriptome and proteome allocation are constant.

a. Transcriptome allocation to 13 gene categories (as defined by Metzel-Raz *et al.*, 2017 [4]) were compared between N-limited and C-limited cultures. Dashed lines are 2-fold increase/decrease from y=x, solid line. Mean values of biological duplicates are shown.
b. As in (a) for proteome allocation.

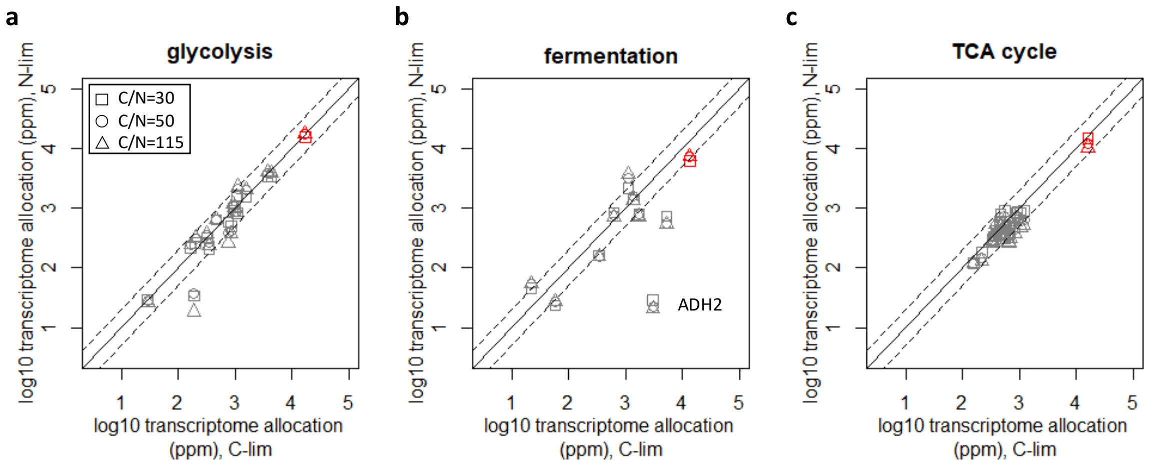

Supplementary Fig. 8. Transcriptomic allocation to core CCM pathways are constant.

a. Transcriptome allocation to each enzyme in glycolysis were compared between N-limited and C-limited cultures. Total transcriptome allocation to all enzymes in glycolysis is shown in red. Dashed lines are 2-fold increase/decrease from y=x, solid line. Mean values of biological duplicates are shown.
b. As in (a) for transcriptome allocation to enzymes in fermentation.
c. As in (a) for transcriptome allocation to enzymes in the TCA cycle.

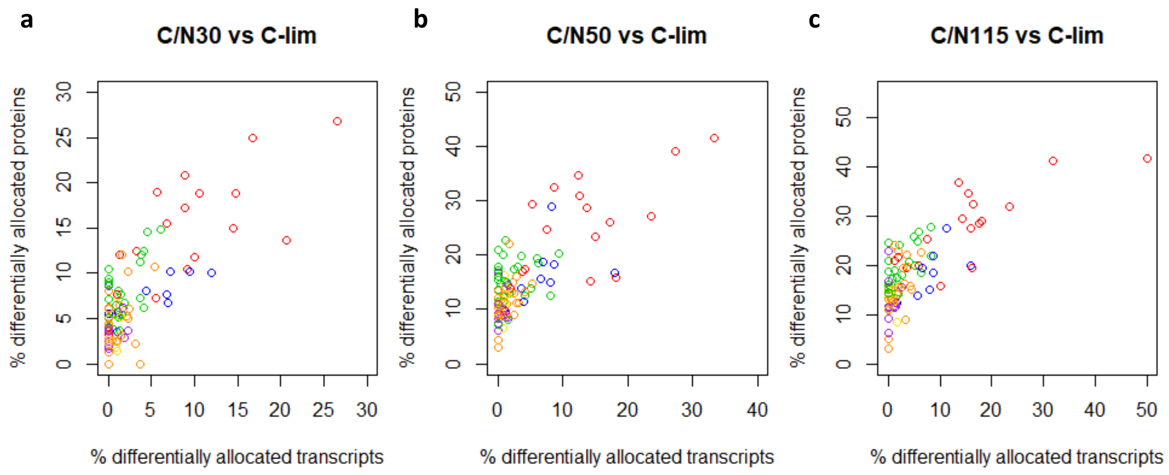

Supplementary Fig. 9. Genes in metabolic processes are more likely to be differentially allocated. a-c. proportion of genes in 99 GO-slim terms that are differentially allocated by >2-fold in the proteome and transcriptome, when comparing N-limited to C-limited cultures. GO-slim terms are color-coded by functional category as in Fig 1b.

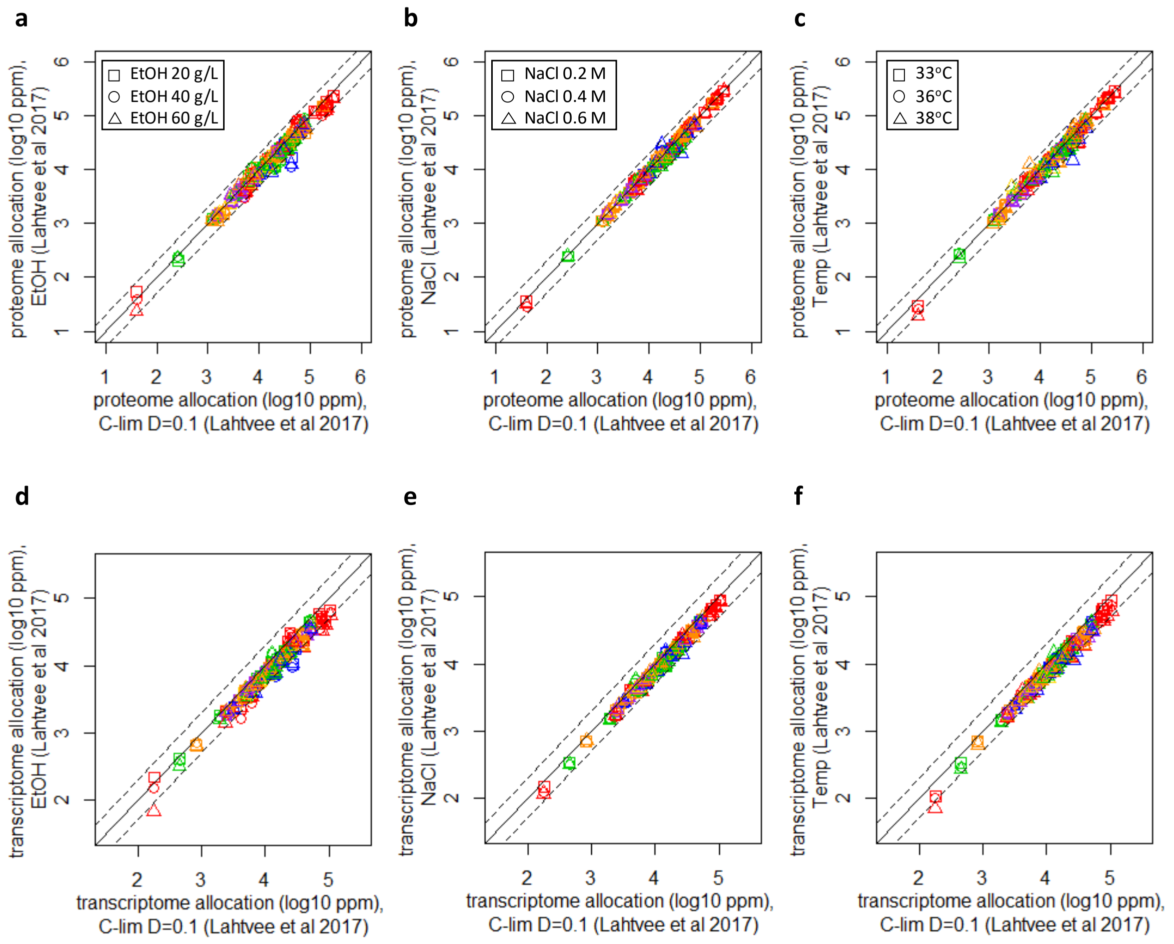

Supplementary Fig. 10. Proteomic and transcriptomic allocation are constant to the growth rate in an independent dataset.

a-c. Proteome allocation to 99 GO-slim terms were compared between stress conditions and reference chemostats as indicated. GO-slim terms are color-coded by functional category as in Fig 1b. Dashed lines are 2-fold increase/decrease from y=x, solid line. Mean values of biological triplicates are shown. ppm, parts per million (mol/mol).

d-f. As in (a-c) for transcriptome allocation.

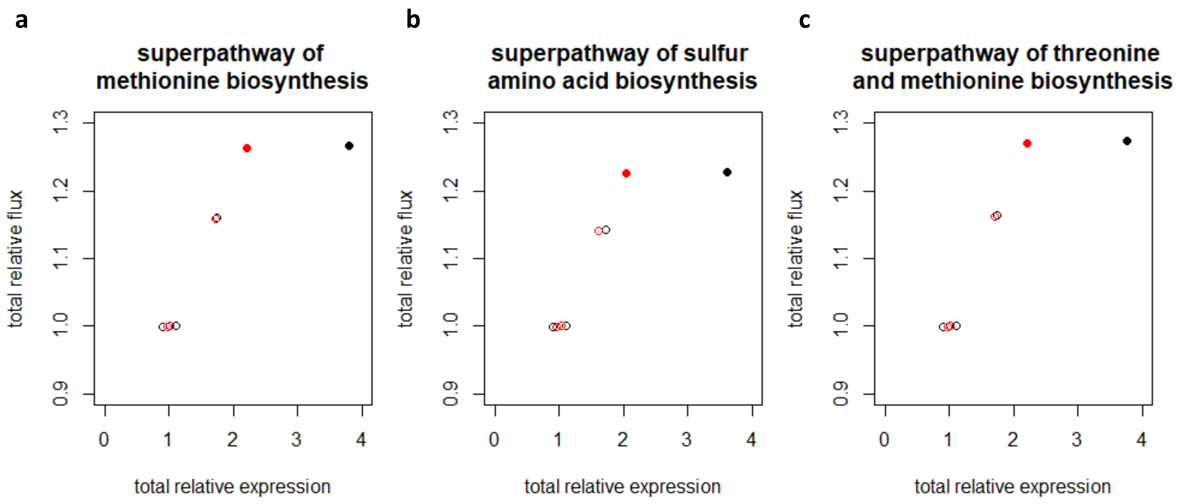

Supplementary Fig. 11. Expression change in MET17 accounts for a large fraction of reserves in the metabolic pathways it participates in.

a-c. the total relative flux and enzyme expression of 3 superpathways with (black) or without (red) MET17 being included in the sum. Open circle, N-limiting conditions. Closed circle, C-limiting condition.

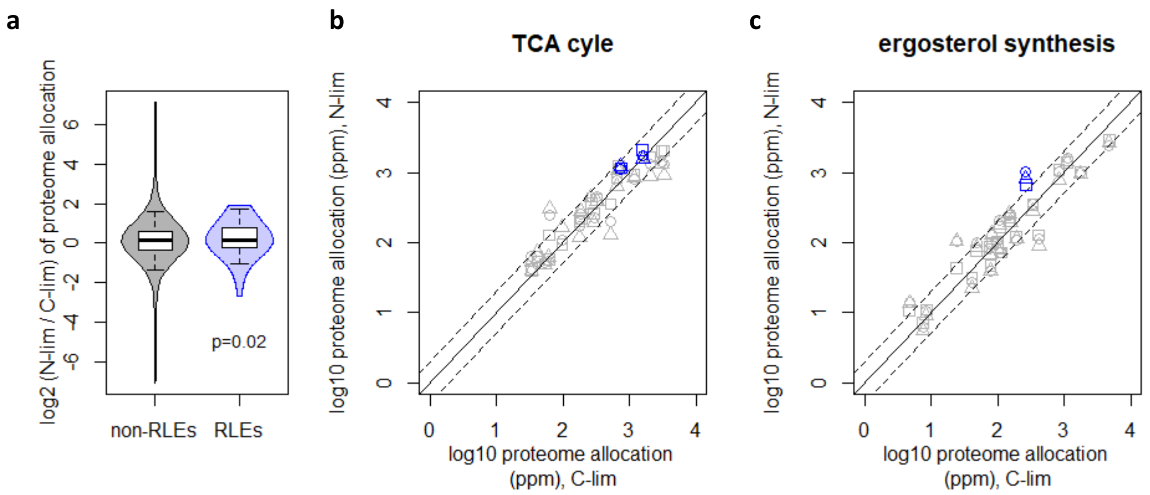

Supplementary Fig. 12. Rate-limiting enzymes (RLEs) and non-RLEs are slightly differentially allocated between C-limited and N-limited conditions.

a. RLEs have a slightly but significant higher proteome allocation in N-limited conditions than non-RLEs. The *p* value indicated is from two-sided Student's t-test. Center line, median; box limits, upper and lower quartiles; whiskers, 1.5x interquartile range.
b. the proteome allocation of metabolic enzymes in the TCA cycle were compared between C-limited and N-limited cultures. RLEs (IDH1 and IDH2) are shown in blue; non-RLEs are shown in grey. Dashed lines are 2-fold increase/decrease from y=x, solid line. Mean values of biological duplicates are shown.
c. as in (a) for enzymes in ergosterol synthesis pathway. The RLE (blue) is ERG20.

a

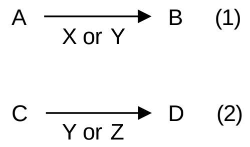

b

	Reactions (1,2,…)	Isozymes (X,Y,Z,…)
Total # in Yeast8.1	173	380
# with >1 isozymes detected	118	238
# with >1 DE isozymes (FC>2)	54	65

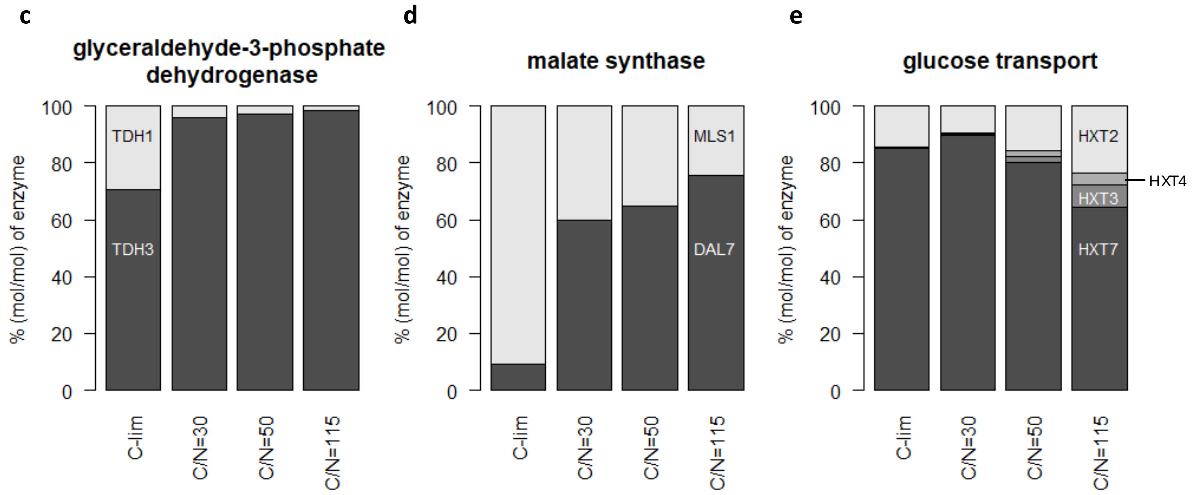

Supplementary Fig. 13. Differential expression of isozymes in C-limited and N-limited cultures.

a. Schematic for reactions catalyzed by isozymes.
b. Number of isozymes detected and differentially expressed (DE) by fold change >2.
c. Differential expression of isozymes for glyceraldehyde-3-phosphate dehydrogenase.
d. Differential expression of isozymes for malate synthase.
e. Differential expression of isozymes for glucose transport. HXT5 is also detected but its expression is too small to be shown (<0.5%).

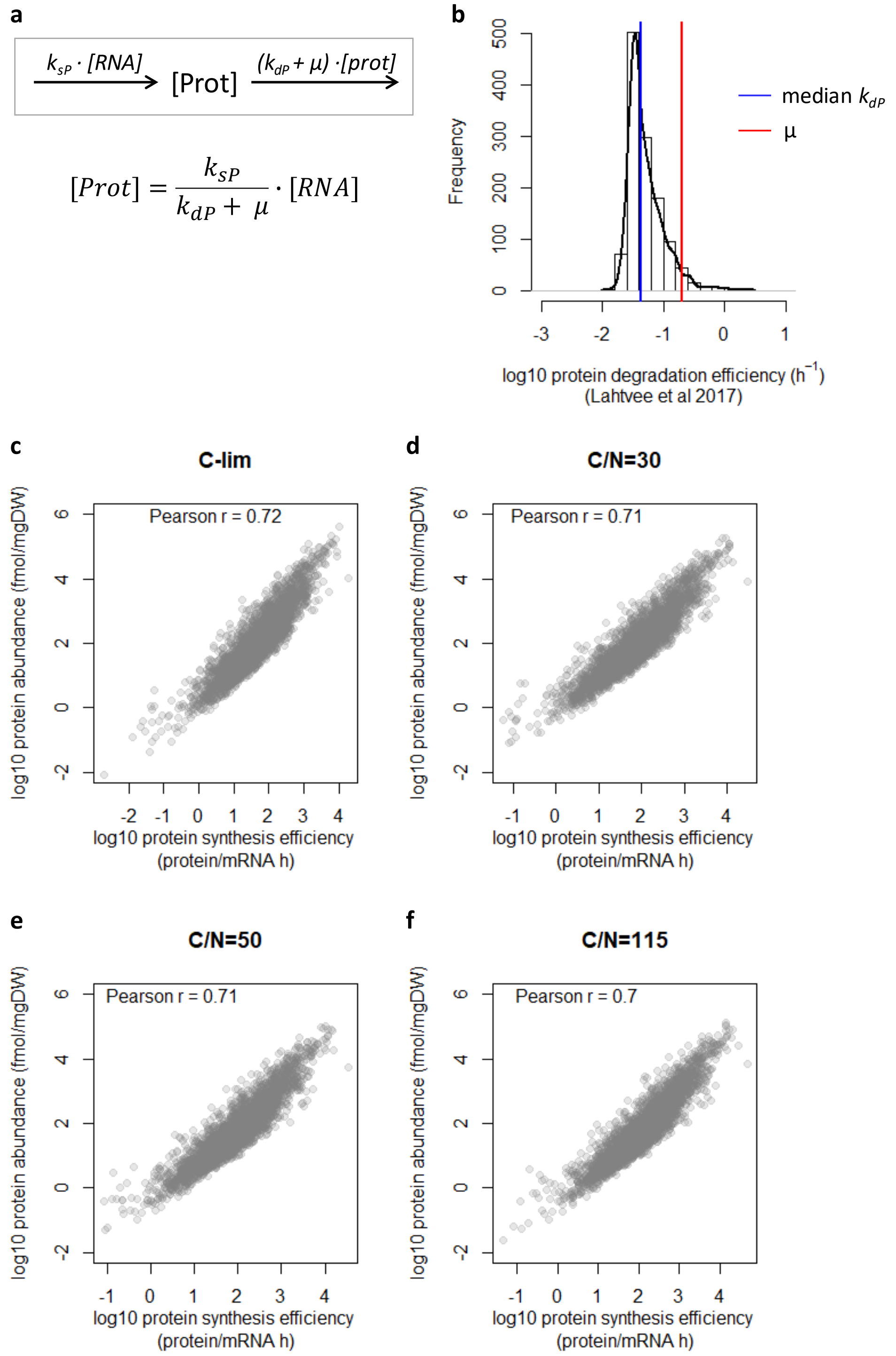

Supplementary Fig. 14. Parameters for calculating protein synthesis efficiency (k_{sP}).

a. Rate equation for the calculation of protein synthesis efficiency (k_{sP}) at steady-state.

b. Protein degradation efficiency was mined from Lahtvee *et al*. (2017) [5]. Blue line indicates the median protein degradation efficiency. The growth rate μ = 0.2 h^{-1} (red line) is shown, demonstrating that μ dominates the (μ + k_{dP}) term in the rate equation.

c-f. Correlation between measured protein abundance and calculated protein synthesis efficiency for each culture condition.

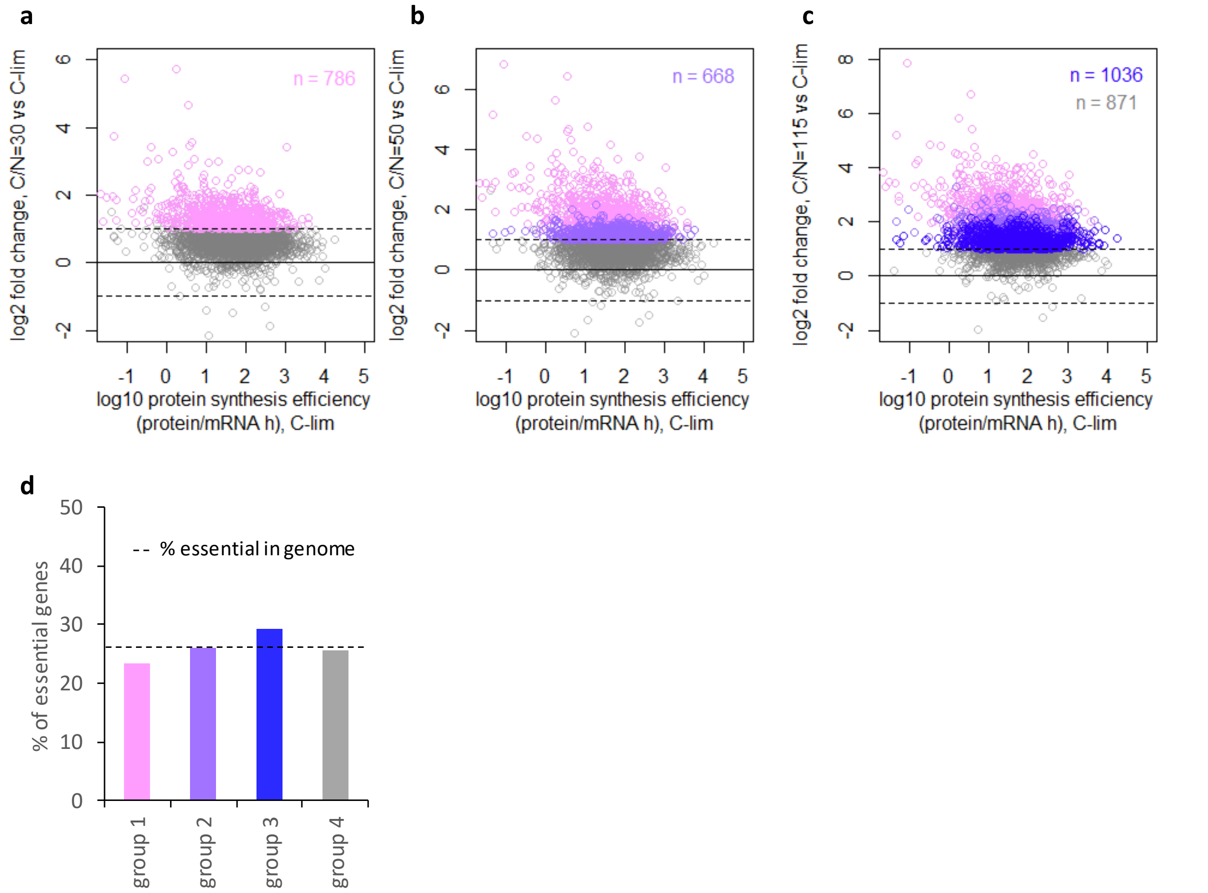

Supplementary Fig. 15. Dynamics of k_{sP} modulation in response to nitrogen reduction.

a. Fold change in k_{sP} of each gene between C/N=30 chemostats and C-limited chemostats were calculated. Genes with >2-fold change in k_{sP} are colored pink.
b. Fold change in k_{sP} of each gene between C/N=50 chemostats and C-limited chemostats were calculated. Genes with >2-fold change in k_{sP} in C/N=50 compared to C-lim chemostats, but not in C/N=30 compared to C-lim chemostats, are colored purple.
c. Fold change in k_{sP} of each gene between C/N=115 chemostats and C-limited chemostats were calculated. Genes with >2-fold change in k_{sP} in C/N=115 compared to C-lim chemostats, but not in C/N=30 or C/N=50 compared to C-lim chemostats, are colored blue.
d. Essential genes were mined from SGD and grouped based on the step of nitrogen reduction at which their translation efficiency was increased by log2 > 1. Bars are color-coded based on gene groups as (a-c). The percentage of genes in each group that are essential was calculated and compared with the percentage of essential genes in the genome (horizontal dashed line). $p_{Fisher} > 0.01$ for all groups.

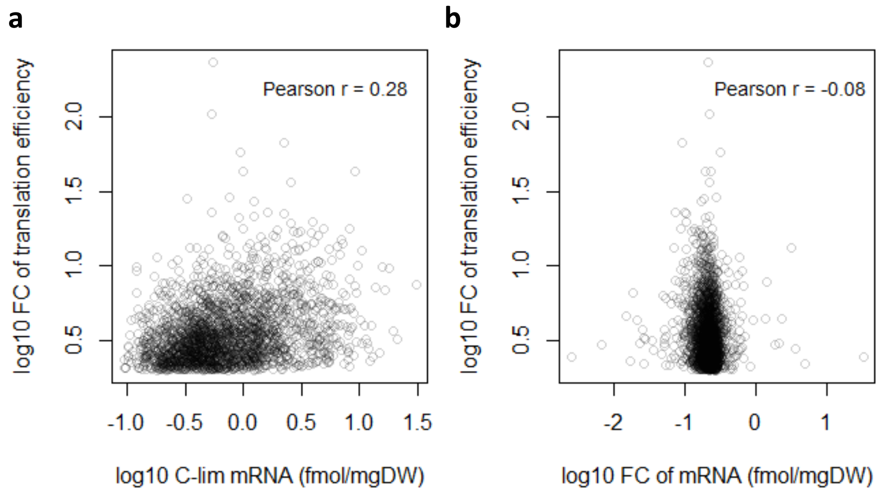

Supplementary Fig. 16. Use of translational reserves is neither correlated with mRNA abundance nor with changes in mRNA abundance between conditions.

a. The change in gene translation efficiency in C/N=115 compared to C-limited cultures, for those genes who engages reserve translational reserves (i.e. all genes except the 871 in the “no modulation” group in Figure 4), is plotted against the abundance of their transcripts in C-limited cultures.
b. The change in gene translation efficiency in C/N=115 compared to C-limited cultures, for those genes who engages reserve translational reserves (i.e. all genes except the 871 in the “no modulation” group in Figure 4), is plotted against the change in abundance of their transcripts in in C/N=115 compared to C-limited cultures.

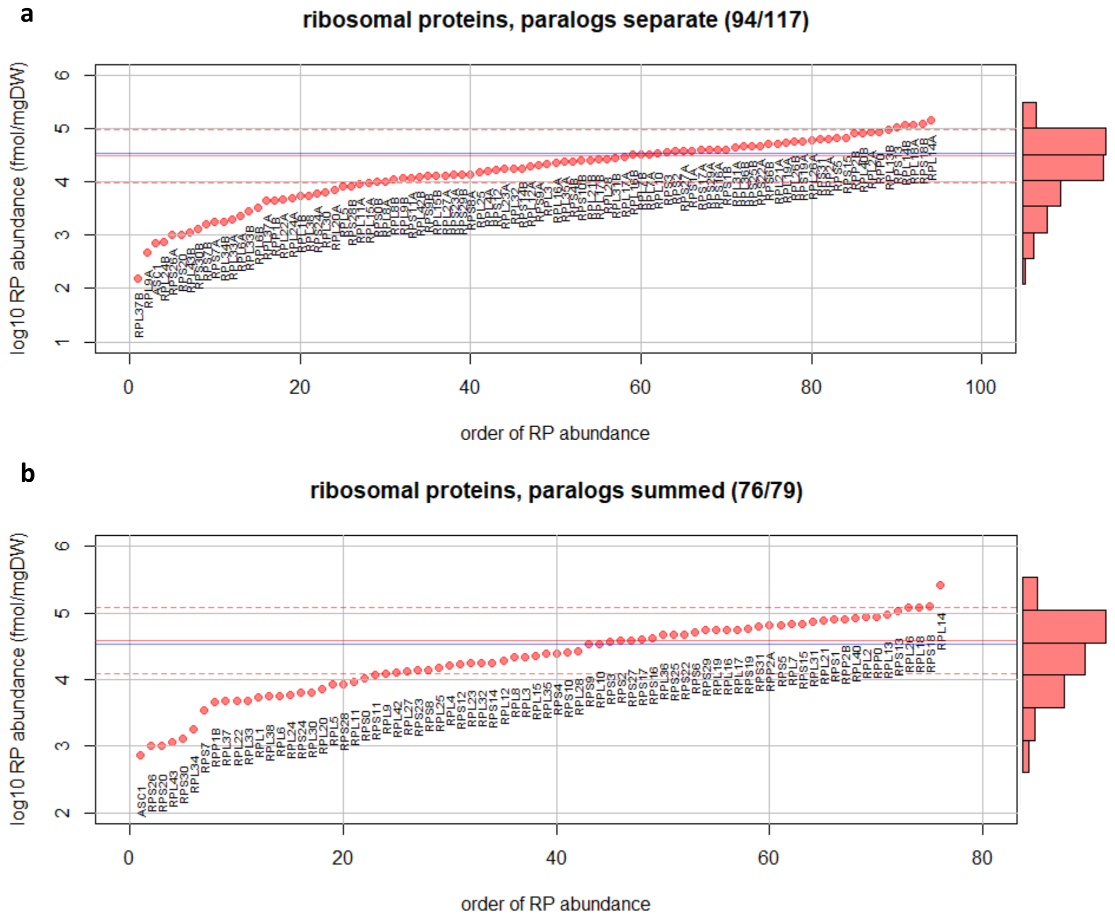

Supplementary Fig. 17. Ribosomal protein stoichiometry deviates from 1:1 in C-limited cultures.

a. The distribution of ribosomal protein (RP) abundance in C-limited chemostats is shown. Red solid line is the mean RP abundance. Blue solid line is the rRNA abundance estimated as 80% of total RNA. Dashed lines are ± log10 of 0.5 from the mean, representing an order of magnitude surrounding the mean. Data points are mean of biological duplicates of each RP.

b. As in (a) with summed paralog abundance.

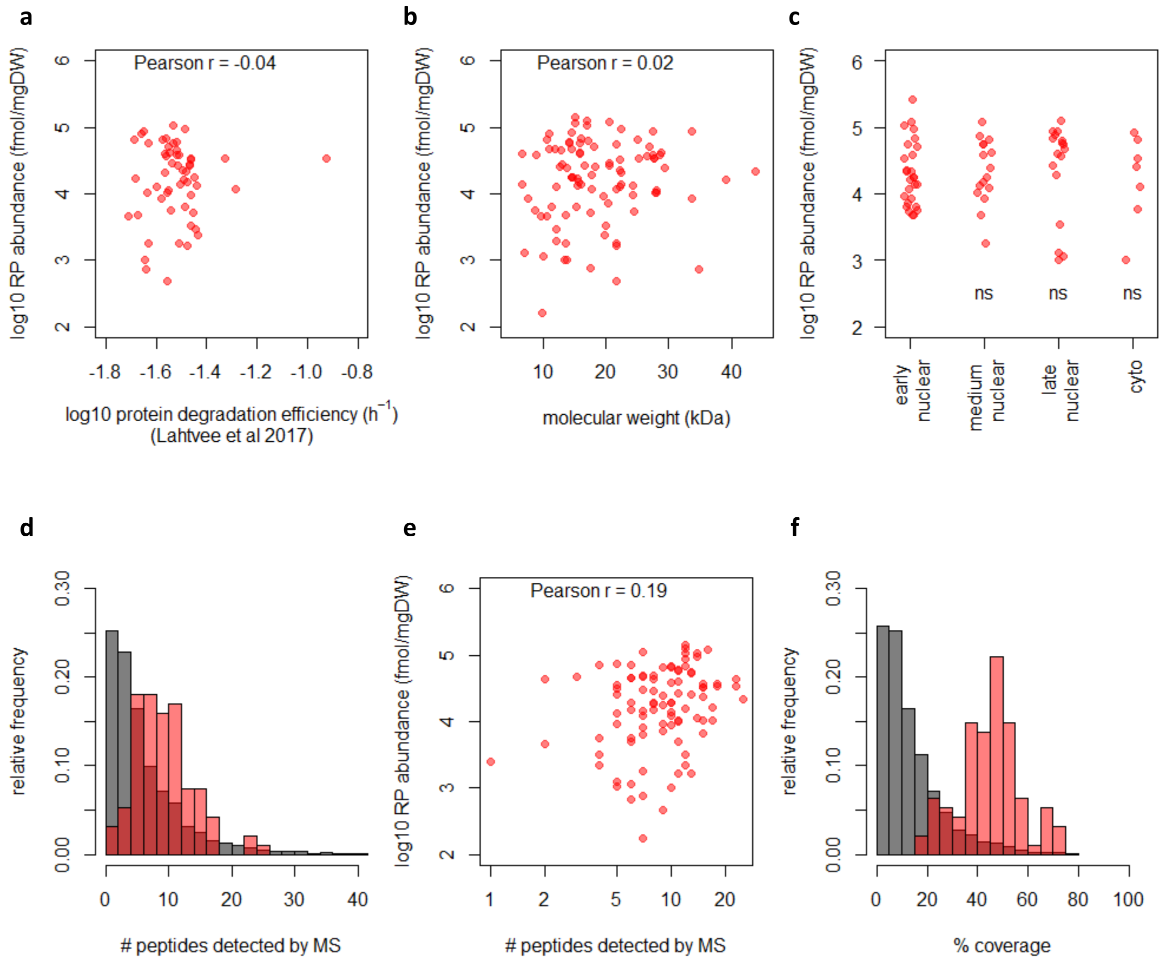

Supplementary Fig. 18. Parameters for the determination of RP abundance stoichiometry.

a. Correlation between RP abundance in C-limited chemostats and protein degradation efficiency mined from Lahtvee *et al* (2017) [5].
b. Correlation between RP abundance in C-limited chemostats and molecular weight.
c. Correlation between RP abundance in C-limited chemostats and order of RP assembly. "Early/medium/late nuclear" represent the first 3 stages of ribosomal assembly in the nucle(ol)us. "Cyto" represents the last stage of ribosomal assembly in the cytoplasm. RP assembly data was mined from de la Cruz *et al.* (2016) [6]. ns, not significant ($p_{Student} > 0.05$).
d. Distribution of # peptides detected by MS. Grey bars indicate all proteins in the dataset; red bars indicate peptides for RPs only.
e. Correlation between RP abundance and # peptides detected by MS is shown.
f. Distribution of % protein sequence covered by MS. Grey bars indicate all proteins in the dataset; red bars indicate peptides for RPs only.

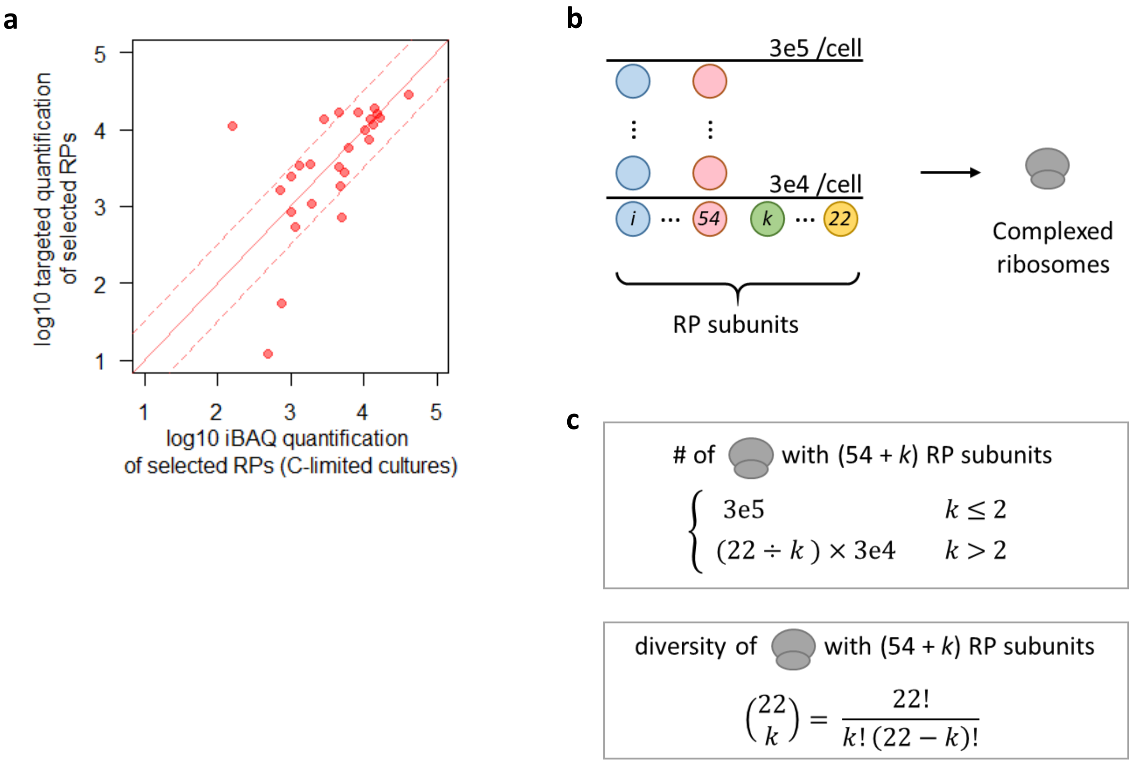

Supplementary Fig. 19. Validation of RP subunit quantification by MS, and modelling of the amount and diversity of complexed ribosomes in the cell.

a. iBAQ-based quantification is robust to the order of magnitude for most RPs in C-limited cultures. Dashed lines are ± log10 of 0.5 from y=x, solid line.

b-c. Schematic and equations for calculating ribosome complex diversity and abundance. *i*, number of RP subunits in the order of 10^5 / cell. *k*, number of RP subunits in the order of 10^4 / cell. Model of ribosome complex diversity and abundance are as Fig 5B-C.

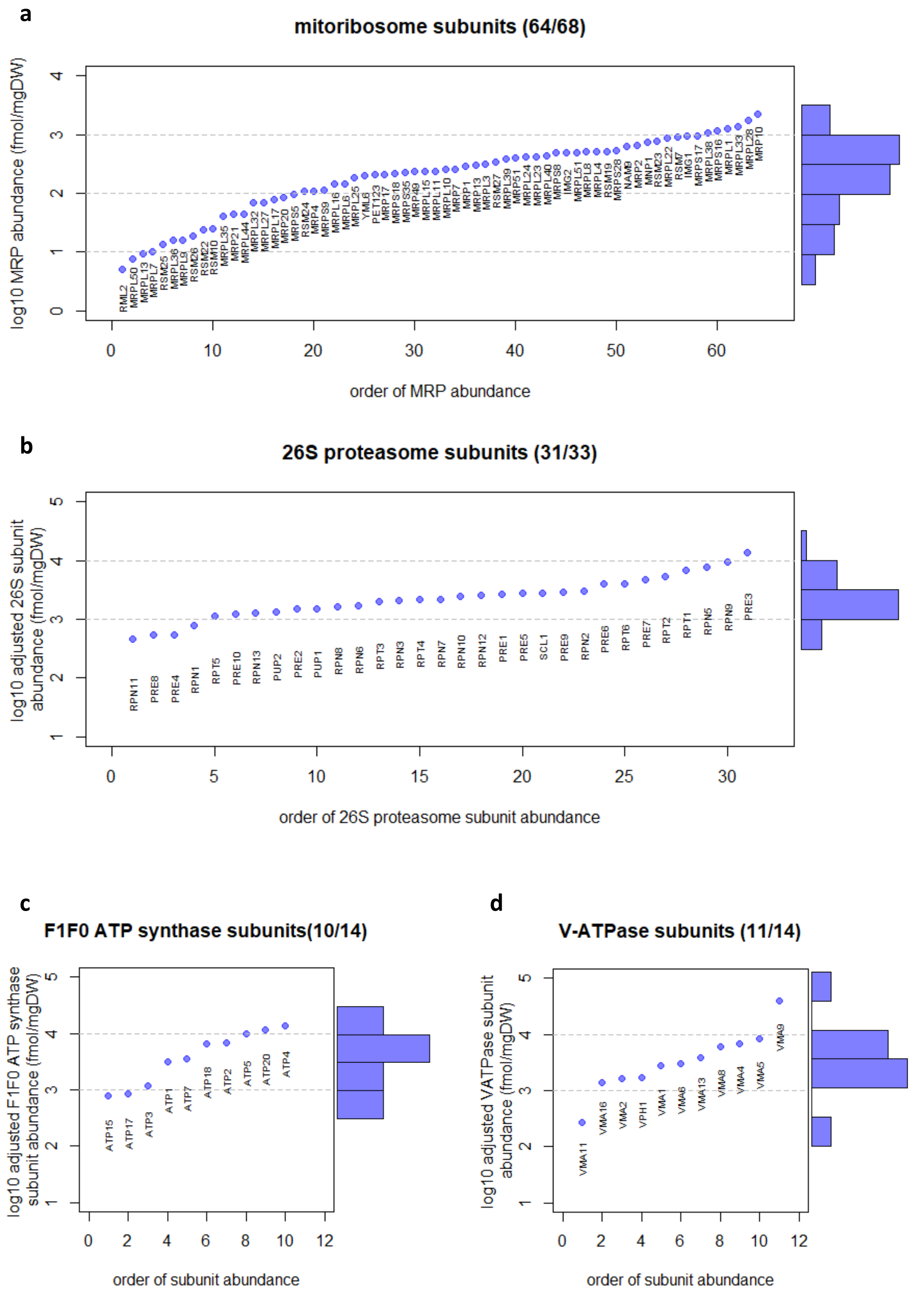

Supplementary Fig. 20. Stoichiometry of selected multi-protein complexes.

a. Mitoribosomal protein subunit abundance in C-limited chemostats is shown. Data points are mean of biological duplicates of each subunit and labeled with standard gene name. Three dashed lines show that subunits span 2 orders of magnitude.
b. As in (a) for 26S proteasome. Two dashed lines show that subunits span 1 order of magnitude.
c. As in (b) for F1F0 ATP synthase.
d. As in (b) for V-ATPase.

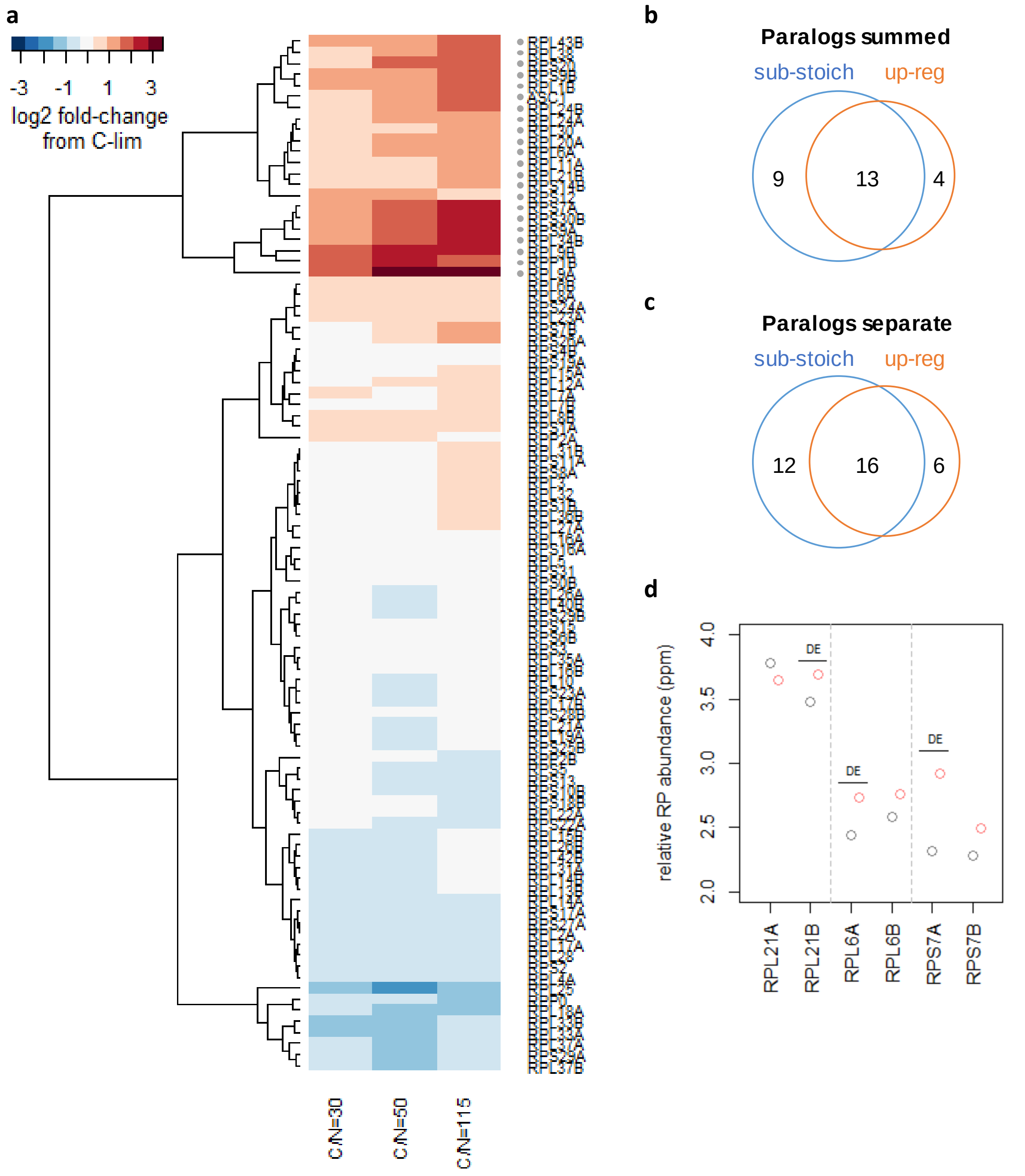

Supplementary Fig. 21. Global modulation of ribosome composition.

a. Relative abundance of RP subunits in N-limited chemostats compared to C-limited chemostat. Paralogs were accounted for separately. The 22 RPs that were selectively upregulated by >2-fold are indicated with a grey dot.

b. Overlap of RP subunits that are sub-stoichiometric in C-limited chemostat (blue), and RP subunits that are upregulated in N-limited chemostats (orange). RP paralogs were summed.

c. As in (b) with paralogs accounted for separately.

d. Three paralog pairs exhibiting differential response under N-limitation. Grey data points are RP abundance in C-limited chemostat. Red data points are RP abundance in N-limited chemostat. DE, differential expression between C-limited and N-limited growth. All data points are mean of biological duplicates.

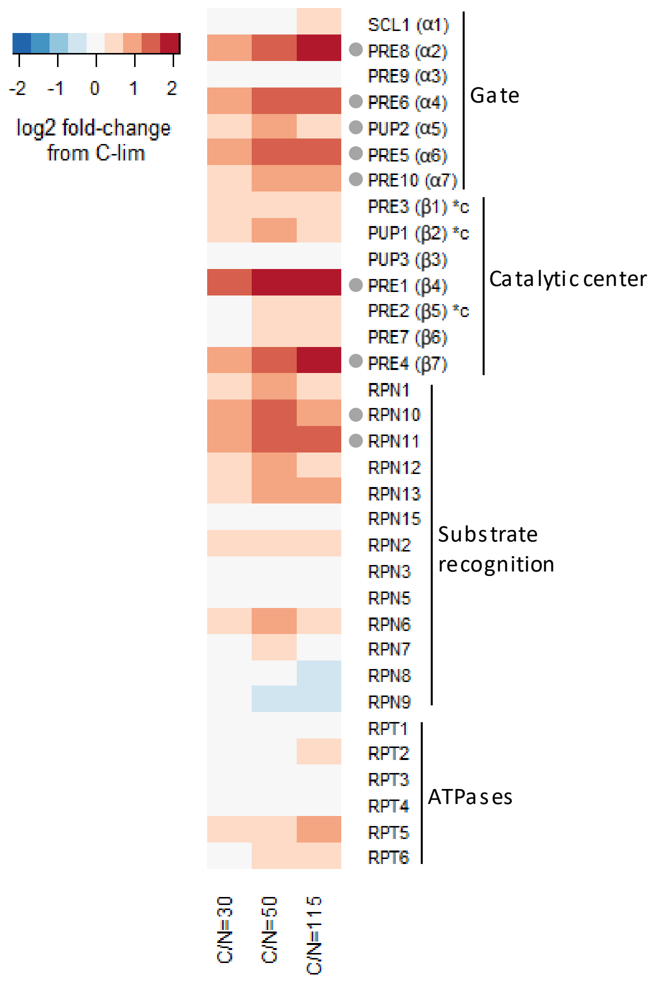

Supplementary Fig. 22. Relative abundance of proteasomal subunits in N-limited chemostats compared to C-limited chemostat. Subunits are course-grain grouped by functional domain. The catalytic subunits are marked with *c. The 9 subunits that were selectively upregulated by >2-fold are indicated with a grey dot.

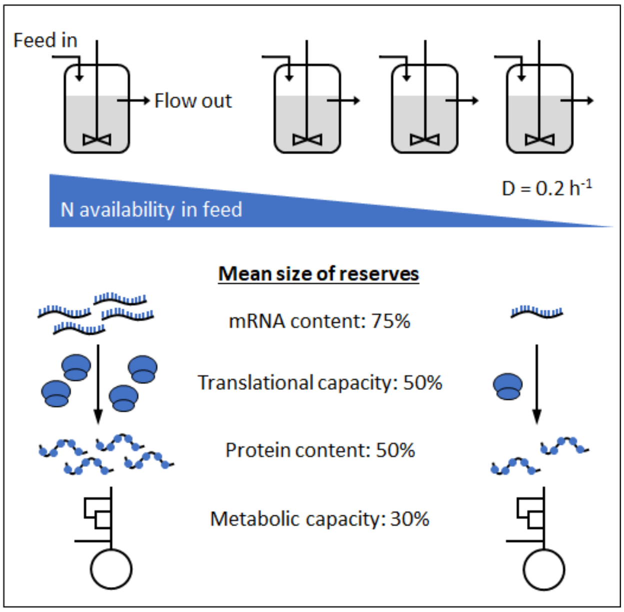

Supplementary Fig. 23. A graphical summary of our major findings.

Supplementary References

1 Schwanhausser, B. *et al.* Global quantification of mammalian gene expression control. *Nature* **473**, 337-342, doi:10.1038/nature10098 (2011).

2 Wisniewski, J. R., Zougman, A., Nagaraj, N. & Mann, M. Universal sample preparation method for proteome analysis. *Nat Methods* **6**, 359-362, doi:10.1038/nmeth.1322 (2009).

3 Perez-Riverol, Y. *et al.* The PRIDE database and related tools and resources in 2019: improving support for quantification data. *Nucleic Acids Res* **47**, D442-D450, doi:10.1093/nar/gky1106 (2019).

4 Metzl-Raz, E. *et al.* Principles of cellular resource allocation revealed by condition-dependent proteome profiling. *Elife* **6**, doi:10.7554/eLife.28034 (2017).

5 Lahtvee, P. J. *et al.* Absolute Quantification of Protein and mRNA Abundances Demonstrate Variability in Gene-Specific Translation Efficiency in Yeast. *Cell Syst* **4**, 495-504 e495, doi:10.1016/j.cels.2017.03.003 (2017).

6 de la Cruz, J., Karbstein, K. & Woolford, J. L., Jr. Functions of ribosomal proteins in assembly of eukaryotic ribosomes in vivo. *Annu Rev Biochem* **84**, 93-129, doi:10.1146/annurev-biochem-060614-033917 (2015).
