# Supplementary material for: Nitrogen limitation reveals large reserves in metabolic and translational capacities of yeast
Source: Nat Commun. 2020 Apr 20;11:1881. doi: 10.1038/s41467-020-15749-0 (PMC7171132; doi:10.1038/s41467-020-15749-0)
Supplement: Supplementary file 3 — Description of Additional Supplementary Files [file 41467_2020_15749_MOESM3_ESM.pdf]

Description of Additional Supplementary Files

File Name: Supplementary Data 1
Description: Absolute protein and mRNA abundances in yeast chemostat cultures.

File Name: Supplementary Data 2
Description: Proteome and transcriptome allocation to different GO-slim processes. Genes are allowed to map to multiple GO-slim terms

File Name: Supplementary Data 3
Description: Detailed breakdown of proteome-transcriptome relationship by 6 functional categories.

File Name: Supplementary Data 4
Description: Measured metabolic flux of yeast chemostat cultures.

File Name: Supplementary Data 5
Description: Proportion of genes in each GO-slim process that are differentially allocated between different conditions.

File Name: Supplementary Data 6
Description: Exchange flux constraints used for flux balance analysis and flux variability analysis.

File Name: Supplementary Data 7
Description: Relative usage of metabolic superpathways. SGD superpathways containing at least 5 genes and with non-zero simulated flux are examined.

File Name: Supplementary Data 8
Description: Model-calculated enzyme exchange and measured enzyme abundance for each enzyme in SGD superpathways containing at least 5 genes and with non-zero simulated flux.

File Name: Supplementary Data 9
Description: Relative isozyme expression. Values represent the % of all isozymes capable of catalyzing the given reaction. Reactions are those in Yeast8.1 that contains an "or" argument in the gene association rule.

File Name: Supplementary Data 10
Description: Protein synthesis efficiency of yeast. "Group" corresponds to Fig 4c; 1=Early modulation; 2=Intermediate; 3=Late modulation; 4=No modulation.

File Name: Supplementary Data 11
Description: Validation of iBAQ-based ribosomal protein quantification by targeted quantitative proteomics.

File Name: Supplementary Data 12
Description: Ribosomal protein abundance, measured by iBAQ and adjusted by targeted quantitative proteomics.
