# Supplementary material for: Nitrogen limitation reveals large reserves in metabolic and translational capacities of yeast
Source: Nat Commun. 2020 Apr 20;11:1881. doi: 10.1038/s41467-020-15749-0 (PMC7171132; doi:10.1038/s41467-020-15749-0)
Supplement: Supplementary file 16 — Reporting Summary [file 41467_2020_15749_MOESM16_ESM.pdf]

nature research

Corresponding author(s): Jens Nielsen

Last updated by author(s): Mar 12, 2020

Reporting Summary

Nature Research wishes to improve the reproducibility of the work that we publish. This form provides structure for consistency and transparency in reporting. For further information on Nature Research policies, see Authors & Referees and the Editorial Policy Checklist.

Statistics

For all statistical analyses, confirm that the following items are present in the figure legend, table legend, main text, or Methods section.

n/a	Confirmed	
☐	☒	The exact sample size (*n*) for each experimental group/condition, given as a discrete number and unit of measurement
☐	☒	A statement on whether measurements were taken from distinct samples or whether the same sample was measured repeatedly
☐	☒	The statistical test(s) used AND whether they are one- or two-sided<br>*Only common tests should be described solely by name; describe more complex techniques in the Methods section.*
☐	☒	A description of all covariates tested
☐	☒	A description of any assumptions or corrections, such as tests of normality and adjustment for multiple comparisons
☐	☒	A full description of the statistical parameters including central tendency (e.g. means) or other basic estimates (e.g. regression coefficient) AND variation (e.g. standard deviation) or associated estimates of uncertainty (e.g. confidence intervals)
☐	☒	For null hypothesis testing, the test statistic (e.g. *F*, *t*, *r*) with confidence intervals, effect sizes, degrees of freedom and *P* value noted<br>*Give P values as exact values whenever suitable.*
☒	☐	For Bayesian analysis, information on the choice of priors and Markov chain Monte Carlo settings
☒	☐	For hierarchical and complex designs, identification of the appropriate level for tests and full reporting of outcomes
☐	☒	Estimates of effect sizes (e.g. Cohen's *d*, Pearson's *r*), indicating how they were calculated

Our web collection on statistics for biologists contains articles on many of the points above.

Software and code

Policy information about availability of computer code

Data collection	Proteome Discoverer 2.2; Mascot 2.5.1
Data analysis	nf-core RNAseq pipeline (available at https://nf-co.re/rnaseq); ecYeast8.1 (available at https://github.com/SysBioChalmers/yeast-GEM); COBRA tool box (available at https://opencobra.github.io/); RAVEN toolbox (available at https://github.com/SysBioChalmers/RAVEN); MATLAB R2016b; R 3.5.1.

For manuscripts utilizing custom algorithms or software that are central to the research but not yet described in published literature, software must be made available to editors/reviewers. We strongly encourage code deposition in a community repository (e.g. GitHub). See the Nature Research guidelines for submitting code & software for further information.

Data

Policy information about availability of data

All manuscripts must include a data availability statement. This statement should provide the following information, where applicable:

- Accession codes, unique identifiers, or web links for publicly available datasets
- A list of figures that have associated raw data
- A description of any restrictions on data availability

Processed quantitative transcriptomics and proteomics data are in Supplementary Table 1. Raw RNA-seq data are available at ArrayExpress, accession E-MTAB-8245 [https://www.ebi.ac.uk/arrayexpress/experiments/E-MTAB-8245/]. The mass spectrometry proteomics data has been deposited to the ProteomeXchange Consortium via the PRIDE 52 partner repository with the dataset identifiers PXD12803 [http://www.ebi.ac.uk/pride/archive/projects/PXD012803] for the IBAQ data set, PXD014962 [http://www.ebi.ac.uk/pride/archive/projects/PXD014962] for the TMT-based relative quantification data set and PXD015025 [http://www.ebi.ac.uk/pride/archive/projects/PXD015025] for the absolute quantification experiment with the standard peptides. GO-slim term are available from the Saccharomyces Genome Database [https://downloads.yeastgenome.org/curation/literature/go_slim_mapping.tab]. All other supporting data are available from the corresponding author on request.

Field-specific reporting

Please select the one below that is the best fit for your research. If you are not sure, read the appropriate sections before making your selection.

☒ Life sciences ☐ Behavioural & social sciences ☐ Ecological, evolutionary & environmental sciences

For a reference copy of the document with all sections, see nature.com/documents/nr-reporting-summary-flat.pdf

Life sciences study design

All studies must disclose on these points even when the disclosure is negative.

Sample size	No sample size calculation was performed. Biological duplicates were used in this study because for multi-omics studies it is quite common to perform them in duplicates and this is generally considered to be sufficient.
Data exclusions	No data were excluded.
Replication	Biological duplicates were used.
Randomization	Randomization was not necessary in study design.
Blinding	Blinding was not applicable in study design.

Reporting for specific materials, systems and methods

We require information from authors about some types of materials, experimental systems and methods used in many studies. Here, indicate whether each material, system or method listed is relevant to your study. If you are not sure if a list item applies to your research, read the appropriate section before selecting a response.

Materials & experimental systems

n/a	Involved in the study	
☒	☐	Antibodies
☒	☐	Eukaryotic cell lines
☒	☐	Palaeontology
☒	☐	Animals and other organisms
☒	☐	Human research participants
☒	☐	Clinical data

Methods

n/a	Involved in the study	
☒	☐	ChIP-seq
☒	☐	Flow cytometry
☒	☐	MRI-based neuroimaging
